# Epidemiology and Clinicopathologic Features with Prognostic Implications of Conventional Ameloblastoma: A 22-Year Retrospective Study

**DOI:** 10.1007/s12105-026-01893-4

**Published:** 2026-02-24

**Authors:** Kittiphoj Tikkhanarak, Nidhi Handoo, John Hellstein, Tamara Busch, Aline Petrin, Erliang Zeng, Hongli Sun, Martine Dunnwald, Azeez Butali

**Affiliations:** 1 Department of Oral Pathology, Radiology and Medicine, College of Dentistry, The University of Iowa, Iowa City, IA USA; 2 Iowa Institute for Oral Health Research, College of Dentistry, The University of Iowa, Iowa City, IA USA; 3 Division of Biostatistics and Computational Biology, College of Dentistry, The University of Iowa, Iowa City, IA USA; 4 Department of Anatomy and Cell Biology, Carver College of Medicine, The University of Iowa, Iowa City, IA USA

**Keywords:** Ameloblastoma, Conventional, Prognosis, Recurrence, Epidemiology, Odontogenic

## Abstract

**Background:**

Ameloblastoma is the most common odontogenic neoplasm of the jaws, characterized by locally aggressive behavior and high recurrence potential. Although its clinicopathologic features have been described in global populations, large-scale institutional data from the United States (U.S.) remain limited. This study aims to characterize the epidemiologic and clinicopathologic features of conventional ameloblastoma over a 22-year period and to identify factors associated with recurrence in cases submitted to a single U.S. academic surgical oral pathology laboratory.

**Methods:**

A retrospective review was conducted of 123 cases of conventional ameloblastoma submitted to the Surgical Oral Pathology Laboratory at the University of Iowa which receives specimens from various states in the U.S. Data included patient demographics, tumor site, radiographic findings, histologic subtypes, treatment modality, and follow-up information. Recurrence-free survival was analyzed using Kaplan-Meier and log-rank tests, and predictors of recurrence were evaluated by Cox proportional hazards modeling.

**Results:**

Among the 123 cases, the mean age was 51 years (range: 11-93), and 56.9% were male. Most tumors involved the mandible (91.9%). Of the 90 cases with available radiographic findings, 54.4% were unilocular, 37.8% multilocular, and 7.8% mixed radiolucent–radiopaque. The most common histologic subtype was follicular admixed with other subtypes (61.8%). Among 39 patients with treatment data, 23 (59.0%) underwent resection, 11 (28.2%) had enucleation and curettage, and 5 (12.8%) received enucleation with peripheral ostectomy. Follow-up information was available for 35 patients, of whom 15 (42.9%) experienced recurrence within 8-360 months. Recurrence-free survival differed significantly by treatment modality (*p* = 0.006), with resection associated with markedly improved outcomes compared with more conservative treatments (adjusted *p* = 0.028).

**Conclusion:**

This study reinforces the importance of surgical treatment selection in the prognosis of conventional ameloblastoma and highlights the need for careful surgical planning to minimize recurrence. Additionally, mixed histopathologic subtypes within the tumor limited the prognostic value of histologic subclassification.

**Supplementary Information:**

The online version contains supplementary material available at 10.1007/s12105-026-01893-4.

## Introduction

Ameloblastoma is the most common true odontogenic neoplasm of the jaws and presents a significant clinical challenge due to its locally aggressive behavior [1]. Despite being histologically benign, it frequently causes extensive jawbone destruction, cortical expansion, and displacement or resorption of adjacent teeth [2]. Clinical management is further complicated by its high recurrence rate, reported in up to 80% of cases treated with conservative approaches [3].

According to the World Health Organization (WHO) Classification of Head and Neck Tumors (5th edition), conventional, unicystic, and peripheral ameloblastoma are recognized as three distinct entities [1]. Among these, conventional ameloblastoma is biologically the most aggressive form, characterized by locally infiltrative growth and a substantially higher risk of recurrence, and higher prevalence compared with unicystic and peripheral ameloblastoma [2].

Epidemiologic studies have demonstrated some geographic variation in the incidence, demographic distribution, and clinicopathologic characteristics of ameloblastoma. Most large-scale analyses originate from Africa and Asia [4–6], whereas data from North America, particularly the United States (U.S.), remain limited. The most recent U.S. study, conducted in the Southeastern region, primarily examined racial and ethnic differences along with clinical features but did not assess prognostic factors or long-term outcomes. Moreover, that study combined both conventional and unicystic ameloblastoma, potentially obscuring disease-specific behavior [7].

Given that conventional ameloblastoma represents the most clinically significant form because of its aggressive local invasion and high recurrence potential [1, 2], the present study focused exclusively on this pathosis. The objectives were to characterize the epidemiologic and clinicopathologic features of conventional ameloblastoma diagnosed over a 22-year period in a U.S. academic surgical oral pathology laboratory in the Midwestern U.S. and to identify prognostic factors associated with recurrence within this population.

## Materials and Methods

This study was approved by the University of Iowa Institutional Review Board (IRB #202504626) and conducted in accordance with federal guidelines and the principles of the World Medical Association Declaration of Helsinki.

### Case Selection

A retrospective review was performed for all cases diagnosed as ameloblastoma between January 1st, 2003 and December 31st, 2024 in the Surgical Oral Pathology Laboratory, University of Iowa College of Dentistry and Dental Clinics. All archived cases diagnosed as “ameloblastoma” were initially retrieved, as this diagnosis in the laboratory records corresponds to conventional ameloblastoma.

Cases were included if they met the histopathologic criteria for conventional ameloblastoma as defined in the WHO Classification of Head and Neck Tumors (5th Edition) [1]. Diagnostic histologic features included nests or strands of odontogenic epithelium lined by peripheral cuboidal to columnar cells with nuclear palisading and hyperchromatic nuclei, exhibiting reverse nuclear polarization that is often less prominent in the plexiform pattern, surrounding central stellate reticulum-like cells, with no cytologic atypia [1].

All microscopic slides were reviewed in conjunction with the clinical and radiographic information provided in the pathology submission forms. Diagnoses were verified by a fourth-year oral and maxillofacial pathology resident (K.T.) and a board-certified oral and maxillofacial pathologist (N.H.). Cases of unicystic ameloblastoma, peripheral ameloblastoma, adenoid ameloblastoma, and ameloblastic carcinoma were excluded. Recurrent lesions were included if the primary diagnosis had been confirmed at the same laboratory or supported by adequate prior documentation.

## Data Collection

Demographic, clinical, and radiographic data were extracted from pathology reports and submission forms. Variables collected included state of specimen submission, patients’ age, sex, and tumor site. Radiographic appearance was based solely on descriptions provided by the referring clinician on pathology submission forms and was categorized as unilocular radiolucency, multilocular radiolucency, or mixed radiolucent-radiopaque.

Histopathologic subtypes were reviewed by K.T. and N.H. and categorized as follicular, plexiform, acanthomatous, granular cell, basal cell, or mixed. A case was classified as one of the primary subtypes when that pattern was predominated in the examined sections. If multiple subtypes were identified, the case was recorded as involving more than one subtype and specified according to the observed components (including follicular with additional subtypes, plexiform with additional subtypes, or follicular and plexiform with additional subtypes). Additional subtypes included acanthomatous, granular cell, desmoplastic, and/or basal cell features. Because follicular and plexiform subtypes are the most common histologic patterns [1], these two were considered the main categories for classification of mixed subtypes. To evaluate intratumoral morphologic diversity, histopathologic heterogeneity was defined by dichotomizing tumors into those exhibiting a single histologic subtype and those showing mixed histologic subtypes within the same lesion.

Treatment information, when available, was classified as resection, enucleation and curettage, or enucleation/excision with peripheral ostectomy. Follow-up data (including recurrence status and time to recurrence) were obtained from pathology submission forms, clinician correspondence, or electronic medical record review within the University of Iowa College of Dentistry and/or the University of Iowa Hospitals and Clinics. The follow-up period was defined as the time from initial surgery to either the date of histologically confirmed recurrence (based on a subsequent pathology submission) or the date of the last documented clinical follow-up with no evidence of recurrence.

### Statistical Analysis

Descriptive statistics were used to summarize demographic, radiographic, histopathologic, and treatment data. Continuous variables (e.g., age and follow-up duration) were summarized as mean ± standard deviation (SD) when normally distributed, and as median with interquartile range (IQR) when non-normally distributed. Comparisons of continuous variables between recurrent and non-recurrent groups were performed based on normality and homogeneity of variance assumptions. If these assumptions were met, Student’s t-test was used; otherwise, the Wilcoxon rank-sum test was applied as a non-parametric alternative. Categorical variables were compared using the chi-square test when all expected cell counts were ≥ 5, or the Fisher’s exact test when expected counts were smaller. Bonferroni correction was applied to adjust for multiple pairwise comparisons of categorical variables. Recurrence-free survival was estimated using the Kaplan-Meier method, and survival differences between groups defined by categorical variables were evaluated using the log-rank test. Holm adjustment was applied for multiple pairwise survival comparisons. Prognostic factors were assessed using the Cox proportional hazards model, and results were reported as hazard ratios (HR) with 95% confidence intervals (CI). A two-tailed *p*-value of < 0.05 was considered statistically significant. All statistical analyses were performed using R software (version 4.4.2; released October 31, 2024).

## Results

A total of 128 cases diagnosed as ameloblastoma were retrieved for review. After histopathologic re-evaluation and assessment of clinical information provided in the biopsy reports, five cases were excluded: three were reclassified as unicystic ameloblastoma, one as peripheral ameloblastoma, and one demonstrated atypical cytologic features (hyperchromatic nuclei and increased mitotic activity) inconsistent with conventional ameloblastoma. Therefore, 123 cases of conventional ameloblastoma were included in the study.

### Distribution of Specimen Submissions by U.S. States

The specimens were predominantly submitted from the Midwestern U.S., accounting for 100 cases (81.3%), with Iowa contributing the largest proportion (55 cases, 44.7%), followed by Wisconsin (16 cases, 13%) and Illinois (13 cases, 10.6%). Additional cases were received from several other states across the U.S. (details provided in Supplementary Table 1).

### Demographics and Anatomic Sites

The mean patient age was 51 years (SD 20.8; range, 11–93). Of the 123 patients, 70 (56.9%) were male and 53 (43.1%) were female. The mandible was the most common site of involvement (113 cases, 91.9%), whereas 10 cases (8.1%) arose in the maxilla. Demographic and anatomic site distributions are summarized in Table 1.

**Table 1:** Demographic, anatomic site, and histopathologic characteristics of conventional ameloblastoma (n = 123)

Variables	Values
*Age (year)*: Mean ± SD (range)	51 ± 20.8 (11–93)
*Sex (number of cases, %)*	
Male	70 (56.9)
Female	53 (43.1)
*Anatomic site (number of cases, %)*	
Mandible	113 (91.9)
Maxilla	10 (8.1)
*Histopathologic subtype (number of cases, %)*	
Follicular with additional subtype(s)*	39 (31.7)
Follicular and plexiform with additional subtype(s)*	23 (18.7)
Follicular	20 (16.3)
Follicular and plexiform	14 (11.4)
Desmoplastic	9 (7.3)
Acanthomatous	8 (6.5)
Plexiform	6 (4.9)
Plexiform with additional subtype(s)*	4 (3.3)
*Histopathologic heterogeniety (number of cases, %)*	
Single subtype	43 (35)
Mixed subtypes	80 (65)

*Additional subtypes including acanthomatous, granular cell, desmoplastic, and/or basal cell subtypes

### Radiographic Appearance

Radiographic information was available for 90 cases. Among these, 49 (54.4%) were unilocular radiolucency, 34 (37.8%) multilocular radiolucency, and 7 (7.8%) demonstrated mixed radiolucent–radiopaque features.

### Histopathologic Subtypes

**Fig. 1:**
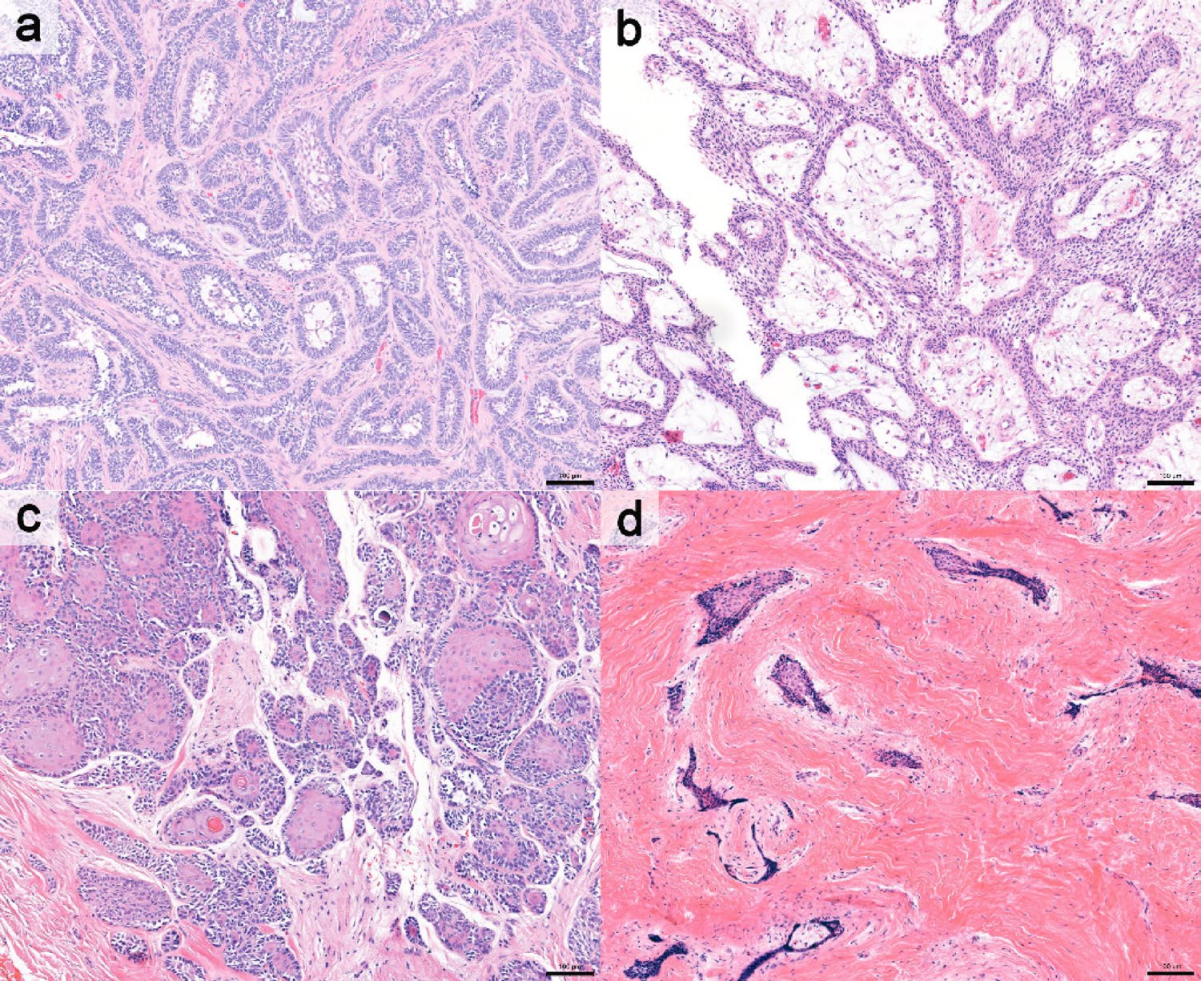
Representative cases showing single predominant histologic subtypes: **a** follicular, **b** plexiform, **c** acanthomatous, and **d** desmoplastic (hematoxylin and eosin [H&E]; scale bar = 100 μm)

**Fig. 2:**
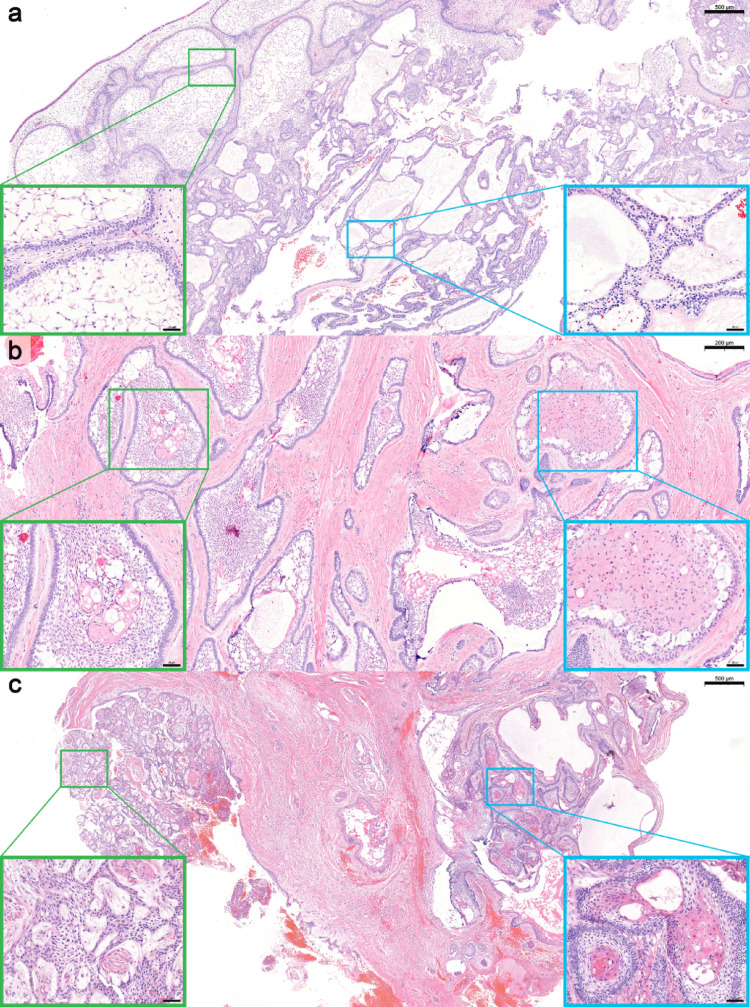
Representative cases of mixed histologic subtypes of conventional ameloblastoma. **a** Mixed follicular and plexiform subtypes (H&E; scale bar = 500 µm); boxed regions corresponding to higher-magnification insets highlighting follicular (green) and plexiform (blue) components (scale bar = 50 µm). **b** Mixed follicular, acanthomatous, and granular cell subtypes (H&E; scale bar = 200 µm); boxed regions corresponding to higher-magnification insets highlighting follicular with acanthomatous (green) and follicular with granular cell (blue) components (scale bar = 50 µm). **c** Mixed follicular, plexiform, and acanthomatous subtypes (H&E; scale bar = 500 µm); boxed regions corresponding to higher-magnification insets highlighting plexiform (green) and follicular with acanthomatous (blue) components (scale bar = 50 µm).

**Fig. 3:**
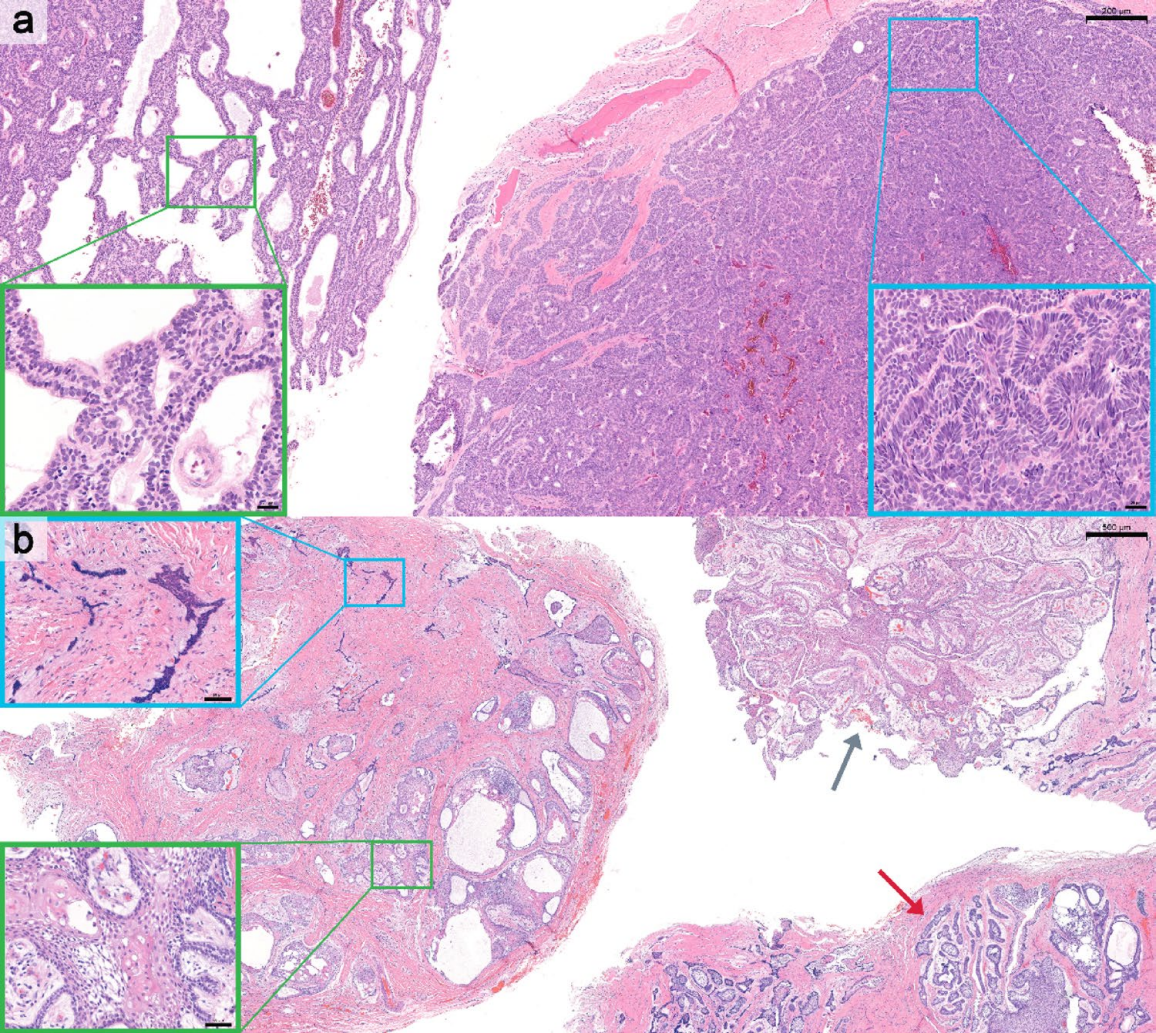
Representative cases of mixed histologic subtypes of conventional ameloblastoma. **a** Mixed plexiform and basal cell subtypes (H&E; scale bar = 200 µm); boxed regions corresponding to higher-magnification insets highlighting plexiform (green) and basal cell (blue) components (scale bar = 20 µm). **b** Mixed follicular, plexiform, acanthomatous, and desmoplastic subtypes (H&E; scale bar = 500 µm). Gray and red arrows indicating regions of plexiform and follicular patterns, respectively. Boxed regions corresponding to higher-magnification insets highlighting follicular with acanthomatous (green) and desmoplastic (blue) components (scale bar = 50 µm)

The predominant histologic subtype was follicular with additional subtype(s) (39 cases, 31.7%), followed by follicular and plexiform with additional subtype(s) (23 cases, 18.7%) and follicular (20 cases, 16.3%). Overall, mixed histopathologic subtypes were identified in 80 cases (65.0%). No cases demonstrated a single predominant granular cell or basal cell subtype. Granular cell and basal cell patterns were identified only as components, occurring in 4 (3.3%) and 7 (5.7%) cases, respectively. Figures 1, 2 and 3 illustrate representative histologic subtypes, and the overall distribution of histopathologic subtypes and heterogeneity is summarized in Table 1. Supplementary Table 2 presents the frequency of individual histopathologic components, irrespective of single predominant or mixed-pattern classification.

### Treatment Modalities

Treatment information was available for 39 patients. Of these, 23 (59.0%) underwent resection, 11 (28.2%) received enucleation and curettage, and 5 (12.8%) underwent enucleation/excision with peripheral ostectomy. Treatment modalities are summarized in Table 2.

**Table 2:** Treatment modalities for conventional ameloblastoma (*n* = 39)

Treatment modality	Number of cases (%)
Enucleation and curettage	11 (28.2)
Enucleation/excision and peripheral ostectomy	5 (12.8)
*Resection* Marginal resection Segmental resection Resection, unspecified	23 (59) − 6 (15.4) − 8 (20.5) − 9 (23.1)

### Clinical Follow-up and Recurrence Outcomes

Follow-up data were available for 35 patients, ranging from 2 to 360 months after surgery (median, 27 months; IQR, 13–65.5). Fifteen patients (42.9%) developed recurrence, whereas 20 (57.1%) remained disease-free. The median follow-up was longer among patients with recurrence (38 months; IQR, 18.5–143) than those without (21 months; IQR, 12.8–64.2), although this difference was not statistically significant (Wilcoxon rank-sum test, *p* = 0.205).

Among the 15 patients who developed recurrence, five had subsequent follow-up information after their first recurrence (range, 6–279 months). Two of these patients experienced a second recurrence. One patient, initially treated with resection, developed a second recurrent ameloblastoma 279 months after undergoing repeat resection for the first recurrence. Another patient, initially treated with enucleation and curettage, developed a second recurrence 111 months after undergoing repeat conservative treatment. The remaining three patients had no additional recurrences, with follow-up durations of 25 and 65 months after resection and 6 months after enucleation/excision and peripheral ostectomy. Supplementary Table 3 provides detailed information for all 35 cases with available follow-up information.

### Factors Associated with Recurrence

Recurrence was significantly associated with younger age (Student’s *t*-test, *p* < 0.001) and treatment modality (Fisher’s exact test, *p* < 0.001). Patients treated with enucleation and curettage had a markedly higher recurrence rate compared with those who underwent resection (pairwise Fisher’s exact test with Bonferroni correction, adjusted *p* < 0.001). There were no significant differences based on sex, anatomic site, radiographic appearance, histopathologic subtype, and histopathologic heterogeneity. Table 3 presents the clinical and histopathologic characteristics and treatment modalities of recurrent and non-recurrent groups.

**Table 3:** Clinical and histopathologic characteristics and treatment approaches of recurrent and non-recurrent groups

Variables	Recurrent (*n* = 15)	Non-recurrent (*n* = 20)	*p*-value
Follow-up period (months); median (IQR)	38 (18.5–143)	21 (12.8–64.2)	0.205 ᵃ
Age (years); mean ± SD	41.1 ± 17.2	56.9 ± 11.9	0.006 ^*,^ ᵇ
Sex, n (%)	Female, 9 (60) Male, 6 (40)	Female, 6 (30) Male, 14 (70)	0.153 ᶜ
Anatomic site, n (%)	Mandible, 15 (100) Maxilla, 0 (0)	Mandible, 17 (85) Maxilla, 3 (15)	0.244 ᵈ
Radiographic appearance^e^, n (%)	Multilocular radiolucency, 6 (50) Unilocular radiolucency, 6 (50)	Multilocular radiolucency, 7 (41.2) Unilocular radiolucency, 7 (41.2) Mixed radiolucent-radiopaque, 3 (17.6)	0.365 ᵈ
Histopathologic subtype, n (%)	Follicular, 6 (40) Follicular with additional subtype(s) ^f^, 6 (40) Follicular and plexiform with additional subtype(s) ^f^, 1 (6.7) Plexiform with additional subtype(s) ^f^, 1 (6.7) Acanthomatous ^f^, 1 (6.7)	Follicular with additional subtype(s) ^f^, 6 (30) Follicular and plexiform, 4 (20) Follicular and plexiform with additional subtype(s) ^f^, 4 (20) Desmoplastic, 2 (10) Follicular, 2 (10) Plexiform, 1 (5) Plexiform with additional subtype(s) ^f^, 1 (5)	0.089 ᵈ
Histopathologic heterogeneity, n (%)	Mixed subtypes, 15 (75) Single subtype, 5 (25)	Mixed subtypes, 8 (53.3) Single subtype, 7 (46.7)	0.329 ᶜ
Treatment modality ^g^, n (%)	Enucleation and curettage, 8 (66.7) Enucleation/excision and peripheral ostectomy, 2 (16.7) Resection, 2 (16.7)	Enucleation/excision and peripheral ostectomy, 1 (5) Resection, 19 (95)	< 0.001 ᵈ

* Statistically significant (*p* < 0.05)

^a^ Wilcoxon rank-sum test; ^b^ Student’s t-test; ^c^ Chi-square test; ^d^ Fisher’s exact test

^e^ Radiographic data available for 12 recurrent and 17 non-recurrent cases

^f^ Additional subtypes including acanthomatous, granular cell, desmoplastic, and/or basal cell subtypes

^g^ Treatment data available for 12 recurrent and 20 non-recurrent cases

### Recurrence-free Survival

**Fig. 4:**
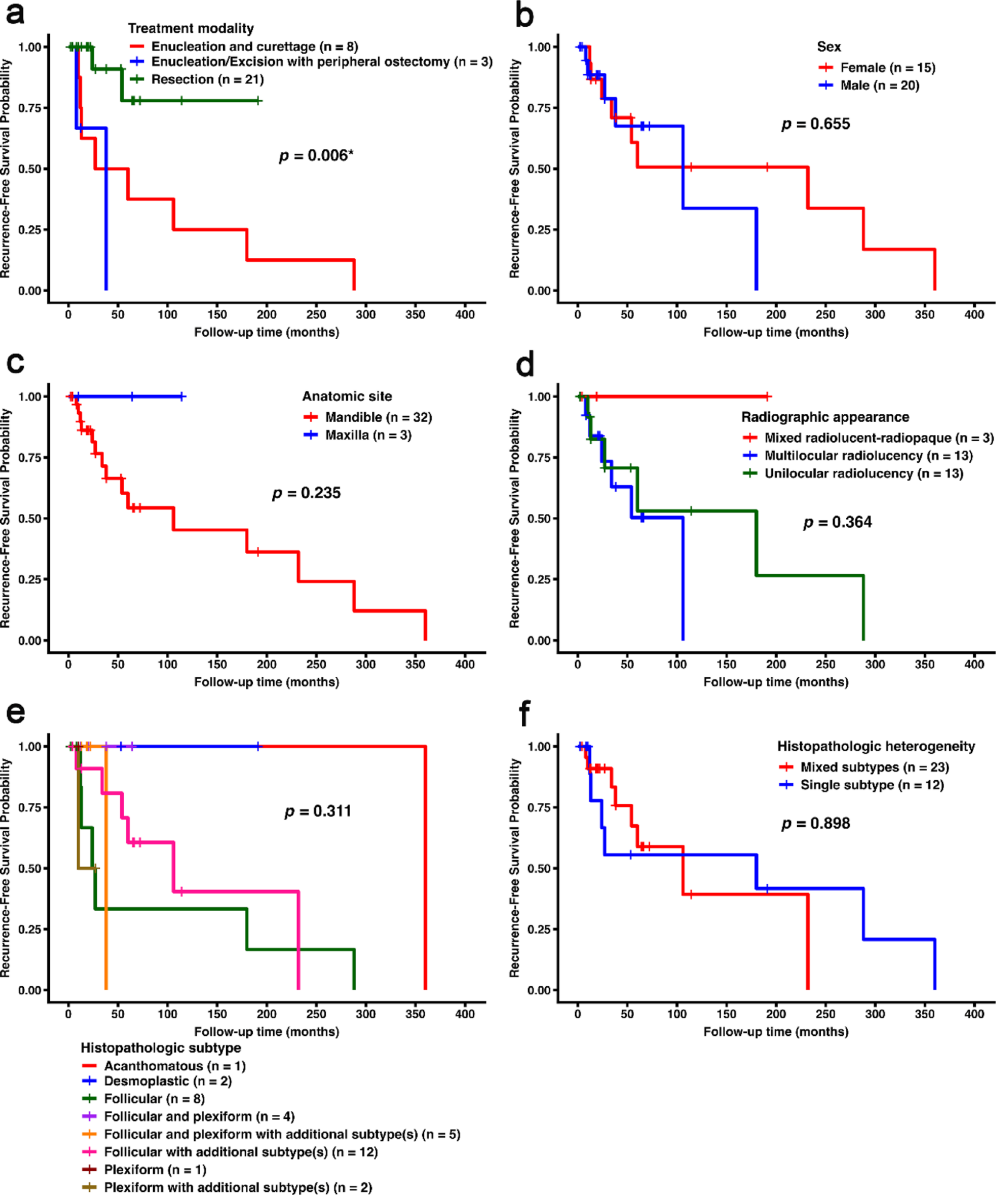
Kaplan-Meier recurrence-free survival curves stratified by **a** treatment modality, **b** sex, **c** anatomic site, **d** radiographic appearance, **e** histopathologic subtype, and **f** histopathologic heterogeneity. *p*-values were obtained from log-rank tests; * indicates statistical significance (*p* < 0.05); Additional subtypes including acanthomatous, granular cell, desmoplastic, and/or basal cell subtypes

Kaplan-Meier survival analysis revealed a significant difference in recurrence-free survival among treatment groups (log-rank, *p* = 0.006; Fig. 4a). Patients treated with resection had the most favorable recurrence-free survival, followed by those treated with peripheral ostectomy and enucleation and curettage. Pairwise log-rank testing with Holm correction confirmed significant differences between resection and enucleation/excision with peripheral ostectomy (adjusted *p* = 0.005) and between resection and enucleation and curettage (adjusted *p* = 0.028). No significant differences in recurrence-free survival were observed based on sex, anatomic site, radiographic appearance, histopathologic subtype, or histopathologic heterogeneity (Fig. 4b–f).

In univariate Cox regression, resection was associated with a significantly reduced hazard of recurrence compared with enucleation and curettage (*p* = 0.028), corresponding to an 83% lower hazard of recurrence. Age, sex, and histopathologic heterogeneity were not significantly associated with recurrence. Table 4 summarizes the results of the univariate Cox regression analyses.

**Table 4:** Univariate Cox proportional hazards regression analysis for recurrence-free survival

Variable	Category	HR	95% CI	*p*-value
Age	–	0.99	0.95–1.02	0.394
Sex	Male	1.30	0.41–4.13	0.656
	Female (ref)	–	–	–
Histopathologic heterogeneity	Single subtype (ref)	–	–	–
	Mixed subtypes	1.08	0.33–3.53	0.898
Treatment modality	Enucleation / curettage (ref)	–	–	–
	Resection	0.17	0.03–0.82	0.028*
	Enucleation/excision and peripheral ostectomy	2.29	0.41–12.66	0.342

*HR* hazard ratio, *CI* confidence interval, *ref* reference category

* Statistically significant (*p* < 0.05)

Other variables, including anatomic site, radiographic appearance, and histopathologic subtype, produced unstable hazard estimates with extremely wide confidence intervals (0–∞) in the Cox regression analysis. This was likely due to sparse data and complete separation, in which recurrence events were present in some categories but absent in others. Therefore, these variables were not further interpreted.

## Discussion

This study provided epidemiologic and clinicopathologic characterization of conventional ameloblastoma over a 22-year period from a single academic surgical oral pathology laboratory in the Midwestern United States (U.S.). To our knowledge, it represents the first institutional analysis from this region. A previous investigation from the Southeastern U.S. focused primarily on racial and ethnic factors [7]. In contrast, our study incorporated detailed treatment information and recurrence-free survival data, providing insight into prognostic patterns and management strategies for ameloblastoma within a U.S. population.

WHO Classification of Head and Neck Tumors (5th edition) classifies conventional ameloblastoma as a distinct entity separate from unicystic and peripheral ameloblastoma [1], supporting the clinical relevance of analyzing this tumor independently. Accordingly, we focused exclusively on conventional ameloblastoma, which demonstrates aggressive local behavior and a substantially greater recurrence risk than unicystic and peripheral ameloblastoma. This approach allowed clearer definition of the clinical and histologic spectrum of the biologically aggressive form of the disease.

Although all specimens were processed at a single reference laboratory, the cohort included submissions from multiple geographical location within the U.S., with nearly half originating outside the state of Iowa. This broad referral base enhances the external validity of our findings, suggesting that the observed patterns are not limited to a single regional population in the U.S. However, this diversity also introduced variability in clinical documentation, radiographic imaging, and treatment approaches, reflecting differences in practice across referring providers and institutions.

The mean patient age in our cohort was 51 years, which aligns with the commonly reported fourth to fifth decade age range for conventional ameloblastoma [1]. This mean age is slightly higher than that reported in a previous U.S.-based study, which documented a mean age of approximately 42 years [7]. A recent global epidemiologic analysis demonstrated that ameloblastoma most commonly occurs in the third decade overall but presents later in Europe and North America, typically in the fifth and sixth decades [5], supporting our findings. A slight male predominance (56.3%) was observed in our study, consistent with reports from global and North American populations, which describe male involvement ranging from 52 to 59% [1, 5].

The mandible was the predominant site of involvement in our cohort (91.9%), consistent with previous U.S. data reporting 93% mandibular involvement [7] and global estimates of 87% [5]. Although a North American systematic review and meta-analysis reported a lower proportion of mandibular cases (71%) [5], the mandible remains the most common site of occurrence across all studies. With respect to specific tumor location, involvement of the posterior mandible is most commonly reported [1]. An exception is the desmoplastic subtype, which has been reported to equally occur in both the mandible and maxilla and shows a greater predilection for the anterior jaws [8]. In our dataset, anatomic subsite information was inconsistently recorded in pathology submission forms, which limited more detailed evaluation of jaw-region involvement.

Radiographically, unilocular radiolucency was the most common presentation in our cohort (54.4%). Although multilocularity has been reported as the predominant pattern in some studies, accounting for up to 74% of cases [1, 9], a multicenter cohort study documented relatively equal frequencies of unilocular and multilocular lesions [4], reflecting variability across studies. Multilocularity was identified as a potential predictor of increased recurrence risk and more aggressive clinical behavior in some studies [10]. In contrast, Bi et al. reported no significant association between radiographic pattern and recurrence risk [11], which is consistent with our findings. Mixed radiolucent-radiopaque radiographic appearance has been described in desmoplastic subtype of conventional ameloblastoma, attributed to reactive bone formation and sclerosis of trabeculae associated with stromal desmoplasia, rather than intrinsic tumor mineralization [1, 8]. A systematic review reported that up to 54% (152 of 279 cases) of conventional ameloblastoma with pure desmoplastic morphology or in combination with other subtypes exhibited mixed radiolucent-radiopaque features [8]. In our cohort, 6 of 24 cases (25%) with desmoplastic component and available radiographic information demonstrated a mixed radiolucent-radiopaque appearance. The predominance of incisional biopsy specimens in our study limited assessment of reactive bone formation in cases demonstrating mixed radiolucent-radiopaque radiographic appearance and may account for the absence of a desmoplastic component in one such case on histologic evaluation. Importantly for radiographic results of our study, radiographic information in our study was derived solely from clinician-provided descriptions on pathology submission forms. As a result, variability in interpretive criteria, imaging modality, and completeness of reporting could not be standardized, and original imaging studies were not available for re-review. Differences between two-dimensional imaging (e.g., panoramic radiograph) and three-dimensional techniques (e.g., cone-beam computed tomograph) may also influence the perception of radiodensity and locularity. In addition, other radiographic features that have been reported to be associated with greater tumor aggressiveness and recurrence risk (e.g., association with impacted teeth, cortical perforation, and root resorption) [10, 11] were not consistently documented in our cohort, limiting further analysis.

Mixed histologic patterns were frequently observed in our cohort, with a predominance of the follicular component combined with other subtypes, consistent with previous studies of ameloblastoma [1, 7]. A recent study from the Southeastern U.S. likewise demonstrated that many ameloblastoma cases exhibited more than one histologic subtype within the same lesion [7], reinforcing the marked histologic heterogeneity of conventional ameloblastoma. These findings underscore the limited prognostic value of subclassifying tumors based solely on histologic subtype. In our analysis, histopathologic subtype did not show a significant difference in recurrence-free survival. A recent scoping review evaluating factors associated with ameloblastoma recurrence exhibited substantial inconsistency across studies regarding the prognostic relevance of histologic subtypes [10]. Much of this variability likely reflects differences in how subtypes were defined across investigations and some studies did not account for tumors exhibiting multiple histologic subtypes within the same lesion [12–14] which is common in our cohort and in previous studies [1, 7, 15]. Furthermore, most specimens in our study were incisional biopsies, which further restrict accurate subtype characterization, as sampling may not capture the full architectural diversity of the lesion. Taken together, the overall evidence supports that histologic subtype alone is not a reliable prognostic indicator in conventional ameloblastoma.

Treatment modality was the significant predictor of recurrence outcome in our analysis. Resection demonstrated substantially improved recurrence-free survival compared with conservative procedures, consistent with previous reports showing lower recurrence rates after radical resection and markedly higher recurrence following conservative treatment [10, 11]. Recurrence occurred as late as 24 and 30 years after initial treatment in patients who were 26 and 33 years old, respectively, at the time of initial diagnosis. Such delayed recurrence likely reflects indolent regrowth of residual tumor following conservative treatment, which is more frequently selected in younger patients to preserve function and quality of life. Notably, two patients experienced recurrence after resection, and one of these developed a second recurrence approximately 23 years after repeat resection. Follow-up periods of 5 to 10 years are commonly reported in the literature [2, 16, 17]; however, our findings in these four cases suggest that long-term postoperative follow-up beyond a decade may be warranted. Recurrence following resection suggests that intrinsic biological factors may contribute to tumor persistence. Molecular alterations involving the MAPK and Hedgehog signaling pathways (particularly BRAF p.V600E and SMO variants) are commonly detected in ameloblastoma and may influence recurrence potential [18–21]. A previous study demonstrated that tumors harboring multiple mutations exhibited a higher recurrence risk compared with those containing a single alteration [22]. Such molecular heterogeneity may help explain why some tumors recur despite aggressive management.

Although younger age appeared to be associated with recurrence in the direct comparison between the two groups (Student’s t-test), this association did not persist in Cox regression analysis, indicating that age was not an independent predictor of recurrence. The significant age difference observed in the t-test likely reflects confounding by treatment modality rather than a biologic effect. Among patients who experienced recurrence, 66.7% underwent enucleation and curettage, whereas 95% of non-recurrent cases were treated with resection. Overall, this marked imbalance suggests that the apparent association between younger age and recurrence is driven by differences in surgical treatment, which is the primary determinant of recurrence in conventional ameloblastoma.

The limitations of this study included variability of follow-up durations. Several patients monitored for shorter periods than typically recommended to detect late recurrence, potentially leading to underestimation of the true recurrence rate. Additional limitations included variability in the completeness of clinical and radiographic data, lack of standardized reporting of prognostic features such as tumor size, association with impacted teeth, and cortical perforation [10], as well as reliance on clinician-provided follow-up for externally managed patients. Original radiographic images were not available for review, and radiographic characterization relied on clinician-provided descriptions, which may be subject to variability. Moreover, the predominance of incisional biopsy specimens in this study limited the ability to fully evaluate histopathologic diversity within individual tumors. The limited number of cases with available follow-up data may affect the interpretation and generalizability of recurrence-related analyses.

Future multi-institutional studies incorporating standardized radiographic, histopathologic, and surgical documentation are needed to validate these findings. Molecular investigations may further elucidate tumor biology and refine recurrence risk prediction. Integrating clinical, surgical, and molecular data has the potential to guide more personalized treatment strategies for ameloblastoma.

In conclusion, this 22-year retrospective analysis of conventional ameloblastoma in a U.S. population aligned with global demographic, anatomic trends, and histopathologic features. This study reinforced that treatment approach remained the key factor influencing recurrence-free survival, emphasizing the critical role of comprehensive surgical management in minimizing recurrence. In addition, this tumor frequently exhibited mixed histopathologic subtypes, limiting the prognostic value of histologic subclassification.

## Supplementary Information

Below is the link to the electronic supplementary material.

Supplementary Material 1

## Data Availability

Most of the datasets analyzed during this study are included in this article and its supplementary files. Additional data supporting the findings of this study are available from the corresponding author upon reasonable request.

## References

[1] WHO Classification of Tumours Editorial Board (2023) Head and neck tumours. 5th ed. WHO classification of tumours series. International Agency for Research on Cancer, Lyon (France)

[2] Effiom OA, Ogundana OM, Akinshipo AO, Akintoye SO (2018) Ameloblastoma: current etiopathological concepts and management. Oral Dis 24:307–316. 10.1111/odi.12646 28142213 10.1111/odi.12646

[3] Pogrel MA, Montes DM (2009) Is there a role for enucleation in the management of ameloblastoma? Int J Oral Maxillofac Surg 38. 10.1016/j.ijom.2009.02.018. :807 – 12 10.1016/j.ijom.2009.02.018 19297131

[4] Dhanuthai K, Chantarangsu S, Rojanawatsirivej S, Phattarataratip E, Darling M, Jackson-Boeters L, Said-Al-Naief N, Shin HI, An CH, Hong NT, An PH, Thosaporn W, Lam-ubol A, Subarnbhesaj A (2012) Ameloblastoma: a multicentric study. Oral Surg Oral Med Oral Pathol Oral Radiol 113:782–788. 10.1016/j.oooo.2012.01.011 22668706 10.1016/j.oooo.2012.01.011

[5] Hendra FN, Van Cann EM, Helder MN, Ruslin M, de Visscher JG, Forouzanfar T, de Vet HCW (2020) Global incidence and profile of ameloblastoma: a systematic review and meta-analysis. Oral Dis 26:12–21. 10.1111/odi.13031 30614154 10.1111/odi.13031

[6] Soyele OO, Akinshipo AO, Effiom OA, Omitola OG, Okoh D, Sigbeku O, Butali A, Adeola HA (2019) A multi-centre evaluation of 566 cases of ameloblastoma in Nigeria by the African oral pathology research consortium. Oral Cancer 3:9–15. 10.1007/s41548-019-00018-6

[7] Vila S, Oster RA, James S, Morlandt AB, Powell KK, Amm HM (2025) A retrospective analysis of 129 ameloblastoma cases: clinical and demographical trends from a single institution. J Racial Ethn Health Disparities 12:1612–1620. 10.1007/s40615-024-01993-3 38607614 10.1007/s40615-024-01993-3 PMC11470111

[8] Chrcanovic BR, Gomes CC, Gomez RS (2020) Desmoplastic ameloblastoma: a systematic review of the cases reported in the literature. Int J Oral Maxillofac Surg 49:709–716. 10.1016/j.ijom.2019.11.004 31810564 10.1016/j.ijom.2019.11.004

[9] Zlotogorski-Hurvitz A, Soluk Tekkeşin M, Passador-Santos F, Martins Montalli VA, Salo T, Mauramo M, Kats L, Buchner A, Vered M (2022) Conceptual changes in ameloblastoma: suggested re-classification of a veteran tumor. Oral Dis 28:703–710. 10.1111/odi.13770 33403703 10.1111/odi.13770

[10] Inthong P, Upalananda W, Saepoo J (2024) Factors associated with recurrence of ameloblastoma: a scoping review. Head Neck Pathol 18:82. 10.1007/s12105-024-01686-7 39177897 10.1007/s12105-024-01686-7 PMC11343934

[11] Bi L, Wei D, Hong D, Wang J, Qian K, Wang H, Zhu H (2021) A retrospective study of 158 cases on the risk factors for recurrence in ameloblastoma. Int J Med Sci 18:3326–3332. 10.7150/ijms.61500 34400902 10.7150/ijms.61500 PMC8364459

[12] Milman T, Ying GS, Pan W, LiVolsi V (2016) Ameloblastoma: 25 year experience at a single institution. Head Neck Pathol 10:513–520. 10.1007/s12105-016-0734-5 27272180 10.1007/s12105-016-0734-5 PMC5082058

[13] Bwambale P, Yahaya JJ, Owor G, Wabinga H (2022) Histopathological patterns and biological characteristics of ameloblastoma: a retrospective cross-sectional study. J Taibah Univ Med Sci 17:96–104. 10.1016/j.jtumed.2021.09.007 35140571 10.1016/j.jtumed.2021.09.007 PMC8801468

[14] Fregnani ER, da Cruz Perez DE, de Almeida OP, Kowalski LP, Soares FA, de Abreu Alves F (2010) Clinicopathological study and treatment outcomes of 121 cases of ameloblastomas. Int J Oral Maxillofac Surg 39:145–149. 10.1016/j.ijom.2009.11.022 20045283 10.1016/j.ijom.2009.11.022

[15] Goh YC, Siriwardena B, Tilakaratne WM (2021) Association of clinicopathological factors and treatment modalities in the recurrence of ameloblastoma: analysis of 624 cases. J Oral Pathol Med 50:927–936. 10.1111/jop.13228 34358362 10.1111/jop.13228

[16] Olaitan AA, Arole G, Adekeye EO (1998) Recurrent ameloblastoma of the jaws. A follow-up study. Int J Oral Maxillofac Surg 27, 456 – 60 9869287 10.1016/s0901-5027(98)80037-4

[17] Reichart PA, Philipsen HP, Sonner S (1995) Ameloblastoma: biological profile of 3677 cases. Eur J Cancer B Oral Oncol 31b:86–99. 10.1016/0964-1955(94)00037-5 7633291 10.1016/0964-1955(94)00037-5

[18] Zhao Z, Xiong G, Wang C, Cao W (2025) From pathogenesis to precision medicine: transformative advances in research and treatment of ameloblastoma. Cancer Lett 612:217448. 10.1016/j.canlet.2025.217448 39800213 10.1016/j.canlet.2025.217448

[19] Sweeney RT, McClary AC, Myers BR, Biscocho J, Neahring L, Kwei KA, Qu K, Gong X, Ng T, Jones CD, Varma S, Odegaard JI, Sugiyama T, Koyota S, Rubin BP, Troxell ML, Pelham RJ, Zehnder JL, Beachy PA, Pollack JR, West RB (2014) Identification of recurrent SMO and BRAF mutations in ameloblastomas. Nat Genet 46:722–725. 10.1038/ng.2986 24859340 10.1038/ng.2986 PMC4418232

[20] Brown NA, Betz BL (2015) Ameloblastoma: a review of recent molecular pathogenetic discoveries. Biomark Cancer 7:19–24. 10.4137/bic.S29329 26483612 10.4137/BIC.S29329 PMC4597444

[21] Kurppa KJ, Catón J, Morgan PR, Ristimäki A, Ruhin B, Kellokoski J, Elenius K, Heikinheimo K (2014) High frequency of BRAF V600E mutations in ameloblastoma. J Pathol 232:492–498. 10.1002/path.4317 24374844 10.1002/path.4317 PMC4255689

[22] Gültekin SE, Aziz R, Heydt C, Sengüven B, Zöller J, Safi AF, Kreppel M, Buettner R (2018) The landscape of genetic alterations in ameloblastomas relates to clinical features. Virchows Arch 472:807–814. 10.1007/s00428-018-2305-5 29388014 10.1007/s00428-018-2305-5 PMC5978850

