# Correction: Epidemiology and Clinicopathologic Features with Prognostic Implications of Conventional Ameloblastoma: A 22-Year Retrospective Study

**DOI:** 10.1007/s12105-026-01914-2

**Published:** 2026-06-05

**Authors:** Kittiphoj Tikkhanarak, Nidhi Handoo, John Hellstein, Tamara Busch, Aline Petrin, Erliang Zeng, Hongli Sun, Martine Dunnwald, Azeez Butali

**Affiliations:** 1 Department of Oral Pathology, Radiology and Medicine, College of Dentistry, The University of Iowa, Iowa City, IA USA; 2 College of Dentistry, Iowa Institute for Oral Health Research, The University of Iowa, Iowa City, IA USA; 3 Division of Biostatistics and Computational Biology, College of Dentistry, The University of Iowa, Iowa City, IA USA; 4 Department of Anatomy and Cell Biology, Carver College of Medicine, The University of Iowa, Iowa City, IA USA

**Correction to: Head and Neck Pathology (2026) 20:25** 10.1007/s12105-026-01893-4.

In the original version of this published article the following errors occurred:

1. Table 1 had formatting issues, with the rows misaligned such that all numbers under the “Values” column did not correspond correctly to their respective titles in the first column. The incorrect and correct Table 1 is given below.
2. Figure 3 caption, “Representative cases of mixed histologic subtypes of conventional ameloblastoma. a Mixed follicular and plexiform subtypes (H&E; scale bar = 500 μm); boxed regions corresponding to higher-magnification insets highlighting follicular (green) and plexiform (blue) components (scale bar = 50 μm). b Mixed follicular, acanthomatous, and granular cell subtypes (H&E; scale bar = 200 μm); boxed regions corresponding to higher-magnification insets highlighting follicular with acanthomatous (green) and follicular with granular cell (blue) components (scale bar = 50 μm). c Mixed follicular, plexiform, and acanthomatous subtypes (H&E; scale bar = 500 μm); boxed regions corresponding to higher-magnification insets highlighting plexiform (green) and follicular with acanthomatous (blue) components (scale bar = 50 μm).” appeared incorrectly. The caption from Fig. 2 has been mistakenly used for Fig. 3. The correct Fig. 3 caption is “Representative cases of mixed histologic subtypes of conventional ameloblastoma. a Mixed plexiform and basal cell subtypes (H&E; scale bar = 200 µm); boxed regions corresponding to higher-magnification insets highlighting plexiform (green) and basal cell (blue) components (scale bar = 20 µm). b Mixed follicular, plexiform, acanthomatous, and desmoplastic subtypes (H&E; scale bar = 500 µm). Red and gray arrows indicating regions of plexiform and follicular patterns, respectively. Boxed regions corresponding to higher-magnification insets highlighting follicular with acanthomatous (green) and desmoplastic (blue) components (scale bar = 50 µm)”. The incorrect and correct Fig. 3 captions are given below.
3. In the last sentence of the first paragraph on page 6 in the published version, “(Fig. 4b and f)” should read as “(Fig. 4b–f).”

The original article has been corrected.

Incorrect Table 1.

**Table 1:**
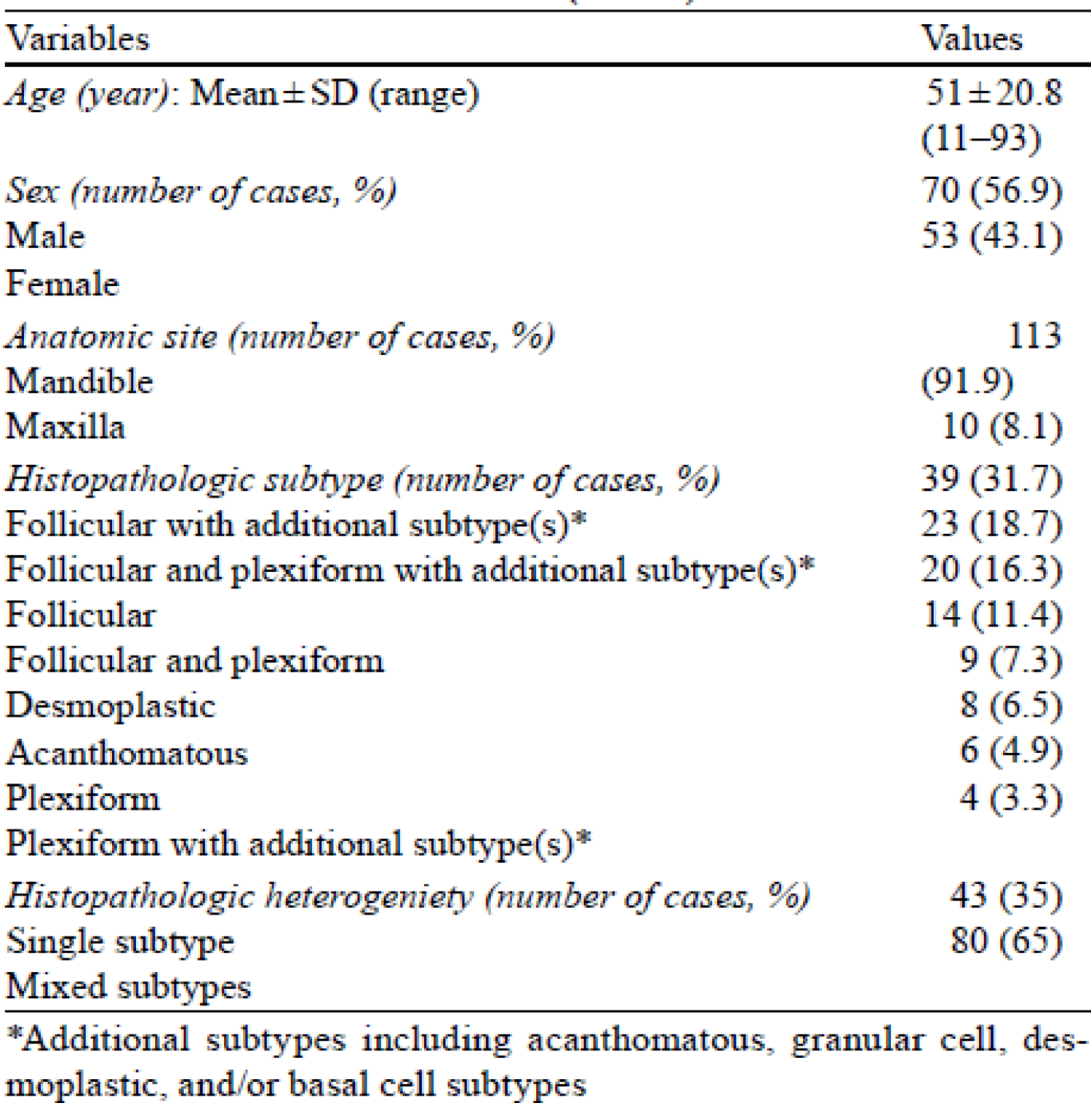
Demographic, anatomic site, and histopathologic characteristics of conventional ameloblastoma (n = 123)

Correct Table 1

**Table 1:** Demographic, anatomic site, and histopathologic characteristics of conventional ameloblastoma (n = 123)

Variables	Values
Age (year): Mean ± SD (range)	51 ± 20.8 (11–93)
Sex (number of cases, %)	
Male	70 (56.9)
Female	53 (43.1)
Anatomic site (number of cases, %)	
Mandible	113 (91.9)
Maxilla	10 (8.1)
Histopathologic subtype (number of cases, %)	
Follicular with additional subtype(s)*	39 (31.7)
Follicular and plexiform with additional subtype(s)*	23 (18.7)
Follicular	20 (16.3)
Follicular and plexiform	14 (11.4)
Desmoplastic	9 (7.3)
Acanthomatous	8 (6.5)
Plexiform	6 (4.9)
Plexiform with additional subtype(s)*	4 (3.3)
Histopathologic heterogenicity (number of cases, %)	
Single subtype	43 (35)
Mixed subtypes	80 (65)

*Additional subtypes including acanthomatous, granular cell, desmoplastic, and/or basal cell subtypes

Incorrect Fig. 3 caption

**Fig. 3:**
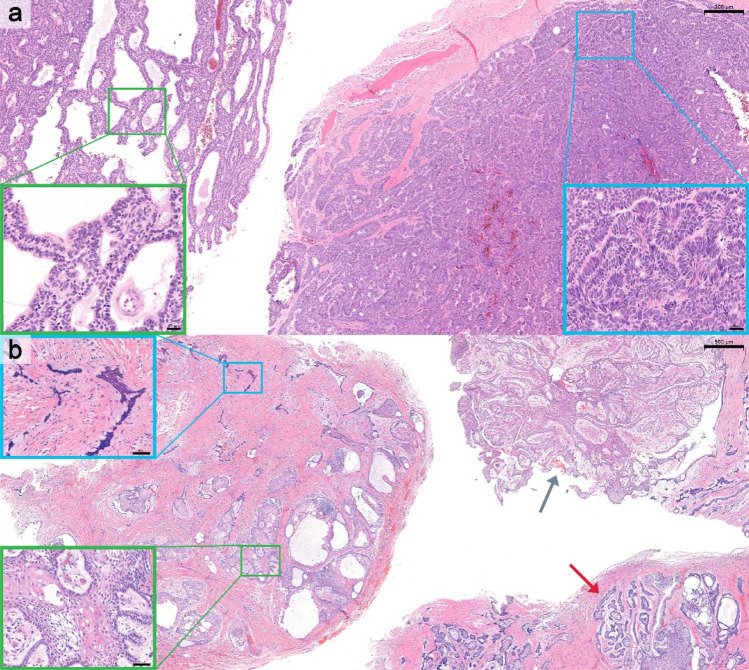
Representative cases of mixed histologic subtypes of conventional ameloblastoma. **a** Mixed follicular and plexiform subtypes (H&E; scale bar = 500 μm); boxed regions corresponding to higher-magnification insets highlighting follicular (green) and plexiform (blue) components (scale bar = 50 μm). **b** Mixed follicular, acanthomatous, and granular cell subtypes (H&E; scale bar = 200 μm); boxed regions corresponding to higher-magnification insets highlighting follicular with acanthomatous (green) and follicular with granular cell (blue) components (scale bar = 50 μm). **c** Mixed follicular, plexiform, and acanthomatous subtypes (H&E; scale bar = 500 μm); boxed regions corresponding to higher-magnification insets highlighting plexiform (green) and follicular with acanthomatous (blue) components (scale bar = 50 μm)

Correct Fig. 3 caption.

**Fig. 3:**
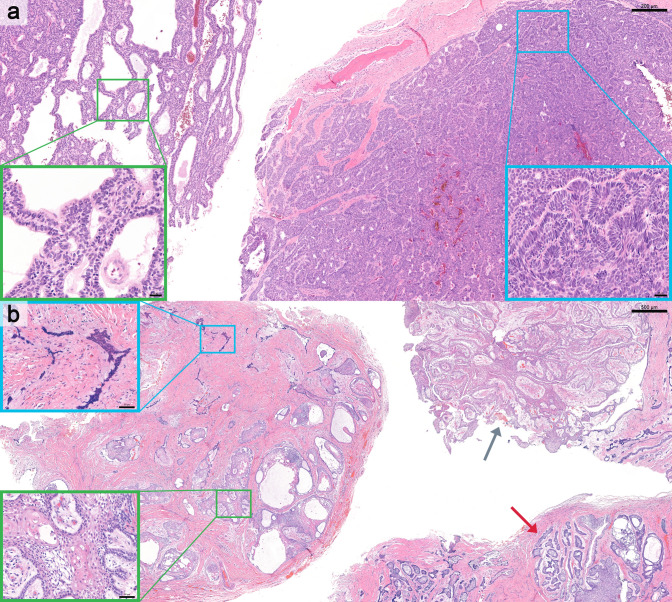
Representative cases of mixed histologic subtypes of conventional ameloblastoma. **a** Mixed plexiform and basal cell subtypes (H&E; scale bar = 200 µm); boxed regions corresponding to higher-magnification insets highlighting plexiform (green) and basal cell (blue) components (scale bar = 20 µm). **b** Mixed follicular, plexiform, acanthomatous, and desmoplastic subtypes (H&E; scale bar = 500 µm). Gray and red arrows indicating regions of plexiform and follicular patterns, respectively. Boxed regions corresponding to higher-magnification insets highlighting follicular with acanthomatous (green) and desmoplastic (blue) components (scale bar = 50 µm)

The original article can be found online at 10.1007/s12105-026-01893-4.

