# Supplementary material for: Epidemiology and Clinicopathologic Features with Prognostic Implications of Conventional Ameloblastoma: A 22-Year Retrospective Study
Source: Head Neck Pathol. 2026 Feb 24;20(1):25. doi: 10.1007/s12105-026-01893-4 (PMC12932794; doi:10.1007/s12105-026-01893-4)
Supplement: Supplementary file 1 — Supplementary Material 1 [file 12105_2026_1893_MOESM1_ESM.docx]

**Supplementary data**

**Supplementary Table 1** Distribution of specimen submissions by U.S. state (n =123)

| **State** | **Number of cases (%)** |
| --- | --- |
| Iowa (IA) | 55 (44.7) |
| Wisconsin (WI) | 16 (13) |
| Illinois (IL) | 13 (10.6) |
| Arizona (AZ) | 10 (8.1) |
| South Dakota (SD) | 10 (8.1) |
| Colorado (CO) | 3 (2.4) |
| Idaho (ID) | 3 (2.4) |
| Minnesota (MN) | 3 (2.4) |
| Hawaii (HI) | 1 (0.8) |
| Kansas (KS) | 2 (1.6) |
| North Carolina (NC) | 2 (1.6) |
| Alabama (AL) | 1 (0.8) |
| Missouri (MO) | 1 (0.8) |
| New Mexico (NM) | 1 (0.8) |
| Utah (UT) | 1 (0.8) |
| Wyoming (WY) | 1 (0.8) |

**Supplementary Table 2** Frequency of histopathologic components identified in conventional ameloblastoma (n = 123)

| **Histopathologic component** | **Number of cases (%)** |
| --- | --- |
| Follicular | 96 (78) |
| Plexiform | 47 (38.2) |
| Acanthomatous | 66 (53.7) |
| Desmoplastic | 27 (22) |
| Granular cell | 7 (5.7) |
| Basal cell | 8 (6.5) |

**Supplementary Table 3** Detailed information for all 35 cases with available follow-up information.

| **Age** | **Sex** | **Site** | **Radiographic appearance** | **Histologic subtype(s)** | **Histologic heterogeneity** | **Treatment modality** | **Follow-up duration (months)** | **Recurrence** | **Second treatment** | **Follow-up duration after first recurrence (months)** | **Second recurrence** |
| --- | --- | --- | --- | --- | --- | --- | --- | --- | --- | --- | --- |
| 26 | F | Mn | Unilocular radiolucency | Follicular | Single subtype | Enucleation and curettage | 288 | Y | Resection, unspecified | 65 | N |
| 66 | M | Mn | Multilocular radiolucency | Follicular with additional subtype(s)* | Mixed subtypes | Enucleation and curettage | 106 | Y | Enucleation/  Excision with peripheral ostectomy | 6 | N |
| 68 | F | Mn | Unilocular radiolucency | Follicular with additional subtype(s)* | Mixed subtypes | Enucleation and curettage | 60 | Y | Enucleation and curettage | 111 | Y |
| 44 | F | Mn | Multilocular radiolucency | Follicular with additional subtype(s)* | Mixed subtypes | Resection, unspecified | 54 | Y | Resection, unspecified | 279 | Y |
| 40 | M | Mn | Unilocular radiolucency | Follicular | Single subtype | Enucleation and curettage | 27 | Y | Segmental resection | 25 | N |
| 26 | F | Mn | Multilocular radiolucency | Follicular | Single subtype | Enucleation and curettage | 12 | Y | NA | NA | NA |
| 47 | M | Mn | Unilocular radiolucency | Plexiform with additional subtype(s)* | Mixed subtypes | Enucleation and curettage | 10 | Y | NA | NA | NA |
| 57 | M | Mn | NA | Follicular and plexiform with additional subtype(s)* | Mixed subtypes | Enucleation/  Excision with peripheral ostectomy | 38 | Y | NA | NA | NA |
| 35 | M | Mn | Multilocular radiolucency | Follicular with additional subtype(s)* | Mixed subtypes | Enucleation/  Excision with peripheral ostectomy | 8 | Y | NA | NA | NA |
| 39 | F | Mn | Multilocular radiolucency | Follicular with additional subtype(s)* | Mixed subtypes | NA | 34 | Y | NA | NA | NA |
| 61 | F | Mn | Unilocular radiolucency | Follicular | Single subtype | Enucleation and curettage | 13 | Y | NA | NA | NA |
| 47 | F | Mn | NA | Follicular with additional subtype(s)* | Mixed subtypes | NA | 232 | Y | NA | NA | NA |
| 14 | M | Mn | Unilocular radiolucency | Follicular | Single subtype | Enucleation and curettage | 180 | Y | NA | NA | NA |
| 13 | F | Mn | NA | Follicular | Single subtype | NA | 24 | Y | NA | NA | NA |
| 33 | F | Mn | Multilocular radiolucency | Acanthomatous | Single subtype | NA | 360 | Y | NA | NA | NA |
| 45 | F | Mx | Unilocular radiolucency | Follicular with additional subtype(s)* | Mixed subtypes | Resection, unspecified | 114 | N | NA | NA | NA |
| 41 | M | Mn | Mixed radiolucent-radiopaque | Follicular and plexiform with additional subtype(s)* | Mixed subtypes | Resection, unspecified | 19 | N | NA | NA | NA |
| 66 | F | Mn | NA | Follicular and plexiform with additional subtype(s)* | Mixed subtypes | Marginal resection | 18 | N | NA | NA | NA |
| 42 | F | Mn | Mixed radiolucent-radiopaque | Desmoplastic | Single subtype | Resection, unspecified | 191 | N | NA | NA | NA |
| 55 | M | Mn | Mixed radiolucent-radiopaque | Follicular with additional subtype(s)* | Mixed subtypes | Marginal resection | 4 | N | NA | NA | NA |
| 43 | F | Mn | Unilocular radiolucency | Desmoplastic | Single subtype | Segmental resection | 53 | N | NA | NA | NA |
| 61 | M | Mn | Unilocular radiolucency | Plexiform with additional subtype(s)* | Mixed subtypes | Segmental resection | 27 | N | NA | NA | NA |
| 66 | M | Mx | NA | Plexiform | Single subtype | Resection, unspecified | 10 | N | NA | NA | NA |
| 68 | F | Mn | Multilocular radiolucency | Follicular and plexiform | Mixed subtypes | Segmental resection | 38 | N | NA | NA | NA |
| 61 | M | Mn | Multilocular radiolucency | Follicular with additional subtype(s)* | Mixed subtypes | Marginal resection | 65 | N | NA | NA | NA |
| 67 | M | Mn | NA | Follicular with additional subtype(s)* | Mixed subtypes | Marginal resection | 72 | N | NA | NA | NA |
| 38 | M | Mn | Multilocular radiolucency | Follicular | Single subtype | Segmental resection | 8 | N | NA | NA | NA |
| 66 | M | Mn | Multilocular radiolucency | Follicular with additional subtype(s)* | Mixed subtypes | Marginal resection | 66 | N | NA | NA | NA |
| 57 | M | Mx | Multilocular radiolucency | Follicular and plexiform | Mixed subtypes | Resection, unspecified | 64 | N | NA | NA | NA |
| 49 | F | Mn | Unilocular radiolucency | Follicular and plexiform with additional subtype(s)* | Mixed subtypes | Segmental resection | 13 | N | NA | NA | NA |
| 47 | M | Mn | Multilocular radiolucency | Follicular and plexiform with additional subtype(s)* | Mixed subtypes | Segmental resection | 22 | N | NA | NA | NA |
| 47 | M | Mn | Unilocular radiolucency | Follicular with additional subtype(s)* | Mixed subtypes | Segmental resection | 13 | N | NA | NA | NA |
| 74 | M | Mn | Unilocular radiolucency | Follicular | Single subtype | Marginal resection | 2 | N | NA | NA | NA |
| 74 | M | Mn | Multilocular radiolucency | Follicular and plexiform | Mixed subtypes | Segmental resection | 20 | N | NA | NA | NA |
| 70 | M | Mn | Unilocular radiolucency | Follicular and plexiform | Mixed subtypes | Enucleation/  Excision with peripheral ostectomy | 12 | N | NA | NA | NA |

**Abbreviations:** M, male; F, female; Mn, mandible; Mx, maxilla; Y, yes; N, no; NA, not applicable.

*Additional subtypes including acanthomatous, granular cell, desmoplastic, and/or basal cell subtypes
